# Intranasal administration of immunogenic poly-epitope from *influenza* H1N1 and H3N2 viruses adjuvanted with chitin and chitosan microparticles in BALB/c mice

**DOI:** 10.22038/IJBMS.2021.58087.12909

**Published:** 2021-08

**Authors:** Sahar Sadeghi, Mojgan Bandehpour, Mostafa Haji Molla Hoseini, Zarin Sharifnia

**Affiliations:** 1 Department of Medical Biotechnology, School of Advanced Technologies in Medicine, Shahid Beheshti University of Medical Sciences, Tehran, Iran; 2 Cellular and Molecular Biology Research Center, School of Advanced Technologies in Medicine, Shahid Beheshti University of Medical Sciences, Tehran, Iran; 3 Department of Immunology, School of Medicine, Shahid Beheshti University of Medical Sciences, Tehran, Iran; 4 Medical Nanotechnology and Tissue Engineering Research Center, Shahid Beheshti University of Medical Sciences, Tehran, Iran

**Keywords:** Chitin microparticles, Chitosan microparticles, Influenza H1N1 virus, Influenza H3N2 virus, Inhaled vaccine

## Abstract

**Objective(s):**

Prevalence of *influenza* virus, creates the need to achieve an efficient vaccine against it. We examined whether the predicted antigenic epitopes of HA, NP, and M2 proteins of the influenza H1N1 and H3N2 viruses accompanied by chitin and chitosan biopolymers might be relevant to the induction of effective proper mucosal responses.

**Materials and Methods:**

The construct was prepared using B and T cell predicted epitopes of HA, NP, and M2 proteins from the influenza H1N1 and H3N2 viruses by considering haplotype “d” as a dominant allele in the BALB/c mice. Intranasal immunization with purified LPS free recombinant protein together with chitin and chitosan microparticles as adjuvants was administered at an interval of 2 weeks in thirty-five BALB/c female mice which were divided into seven groups. Ten days after the last immunization, humoral and cellular immune responses were examined.

**Results:**

Elevated systemic IgG2a, IgA, and mucosal IgA revealed a humoral response to the construct. An increase in the number of IFN-γ-producing cells in re-stimulation of splenocytes in the culture medium by poly-tope as well as rise in the concentrations of IL-6, IL-17, and TNF-α along with the regulatory response of IL-10, presented the capacity of the designed protein to provoke significant immune responses. The neutralization test ultimately confirmed the high efficacy of the protein in inhibiting the virus.

**Conclusion:**

The results support the fact that immunogenic poly-tope protein in the presence of chitin and chitosan microparticles as mucosal adjuvants is able to induce humoral and cell-mediated responses in BALB/c mice.

## Introduction

Vaccination is the most effective way to control and prevent infectious diseases. Despite the existence of licensed inhaled vaccines (FluMist/Fluenz®, Nasovac™), the development of intranasal administration ones, is one of the approaches still being researched in vaccine formulations. Conventional vaccine administration routes for respiratory infections, do not stimulate effective immunity in the upper respiratory tract, so vaccinated individuals can remain carriers of the pathogen without any symptoms. The local pH, mucociliary clearance, and nasal enzymes negatively affect nasal vaccination. To cope with these problems, antigens should be delivered by the proper mucosal adjuvants to keeping them stable in the nasal mucosa, attaching them to the nasal mucus, and by increasing their residence time at mucosal surfaces, and present them more effectively to the APCs (Antigen Presenting Cells). Also the chosen adjuvant should produce an expedient stimulatory pattern without causing harmful effects and allergies. A large number of studies have investigated the potential of biopolymers such as chitin (Poly β-(1-4)-N-Acetyl-D-glucosamine) and chitosan (the chitin de-acetylated derivative) as a delivery system and adjuvant for nasal vaccination. Especially the application of chitin and chitosan microparticles and nanoparticles has been examined (1-7). Chitin and chitosan have been considered as biodegradable and biocompatible non-toxic biopolymers, and their adjuvant properties have been widely studied, mainly together with flu vaccines (8, 9). They have characteristics such as intrinsic immune stimulation and bio-adhesion and can be easily applied in various shapes and sizes (including nanoparticles and microparticles) (10). In addition to vaccine production using conventional methods, nowadays, in the post-genomic era, based on computational resources, several tools have been developed by the use of machine learning systems and statistical methods, which can expedite the conversion of immunological data to meaningful concepts, so the new field named immune-informatics was created. Due to advances in omics fields (genomics, proteomics, transcriptomics, and immunomics) new antigenic targets can be made easily discoverable, so more promised vaccines can be rationally designed by reverse vaccinology and *in silico* tools (11). Epitope mapping is an appropriate approach for virtual screening of amino acid sequences of virulent proteins of pathogens to predict their antigenic and immunogenic regions (epitope) (12, 13). The design and discovery of vaccines for some microorganisms are challenging. One of the main respiratory pathogens, *influenza *virus type A, is attributed to a large burden of contagious respiratory infections worldwide. Despite advances in medical sciences, morbidity and mortality of influenza viruses continue. *influenza* viruses contain four types (A, B, C, and D) which are RNA viruses that belong to the *orthomyxoviridae* virus family (14). They cause respiratory or gut infections in mammals and birds, but in humans, yearly epidemic diseases are caused by A and B virus types (15-17). Every year almost up to five million people are infected by this virus and 290 to 650 thousand deaths occur (18), especially in over 65 elders. Current flu vaccine types are subunit or live-attenuated viruses in the form of nasal spray or injection that are produced every year based on circulating subtypes. Common *influenza *vaccines are strain-dependent so some year mismatch between the viruses in the vaccine and those in circulation occur (19). Flu vaccines mostly trigger a humoral immune response against HA variable regions, but it is well known that clearance of virus and cross-protection in *influenza *infection are mediated by T-cells (20). Generally, current vaccines have only 60% efficacy even by matched ones. High-risk populations like the elderly and patients with specific conditions (diabetics, children, elders, and immune-compromised persons) elicit poor immune responses to present vaccines (21). So, it seems effective immunization strategies and vaccination protocols are needed for this pathogen. Investigations have shown that *in silico* screening of potential *influenza* epitopes can lead to rational design of peptide-based vaccine candidates with the capability of inducing immunogenic responses (22, 23). Polypeptide vaccines cannot create significant immune responses alone (24); and for intranasal administrations, mucosal or respiratory proper adjuvants should be used (25). Although several clinical trials have been conducted on inhaled vaccines, no poly-epitope vaccines have yet been introduced to the market (26-28). In this study, we chose hemagglutinin, nucleoprotein, and matrix protein 2 by consideration of their function and role during infection by *influenza *virus types A H1N1 and H3N2 and designed a new poly-epitope construct by linking their individual predicted T and B cell epitopes by flexible and immune system stimulator linkers. Our construct named IVP accompanied with micro-particles as mucosal adjuvants showed acceptable induced humoral and cellular immune responses in BALB/c mice. The results of neutralization assays showed their capacity to inhibit the virus. To achieve an efficient vaccine for *influenza* virus type A, immunological responses were induced after intranasal administration of the computationally designed poly-epitope along with chitin and chitosan microparticles as potent mucosal adjuvants. Neutralization tests confirmed effective immune responses in inhibiting *the influenza* virus.

## Materials and Methods

***Antigen and adjuvant selection***

The target proteins were chosen from *influenza *H1N1 and H3N2 viruses based on immunogenicity in various literature (14, 17, 29–33)a more profound knowledge of the virulence factors responsible for the morbidity and mortality caused by Streptococcus pneumonia is necessary. This review deals with the major structures of pneumococci involved in the pathogenesis of pneumococcal disease and their interference with the defense mechanisms of the host. It is well known that protection against S. pneumoniae is the result of phagocytes of invading pathogens. For this process, complement and anticapsular polysaccharide antibodies are required. Besides, relatively recent experimental data suggest that protection is also mediated by the removal of disintegrating pneumococci and their degradation products (cell wall, pneumolysin. We considered three efficient proteins in virus infection. Hemagglutinins (HA) are trimer proteins on the surface of *influenza* viruses. Nucleoprotein (NP) is a component of the ribonucleoprotein (RNP) complex. M2 protein, is a transmembrane proton channel. It is a genetically and functionally conserved protein across *the influenza *type A virus. We applied small-sized chitin and chitosan microparticles according to the previous experiments (7-9) as adjuvant candidates. 

***Immune-informatics Studies***

*Proteins sequences retrieval*

Complete amino acid sequences of proteins, retrieved from the UniProtKB database, in FASTA format (Table 1).

*Epitopes prediction*

By considering haplotype “d” as a dominant allele in the BALB/c mice population we predicted MHC I and II restriction epitopes in the IEDB server (Immune Epitope Database and Analysis Resources) (www.iedb.org)(34). The dominant HLA allele was selected by Allele Frequency Net Database( http://www.allelefrequencies.net/) server. Nine-mer long epitopes that are more frequent than 8, 10, or 11-mer peptides were selected in the algorithm. T-cell epitopes were determined among MHC I and II binding predicted peptides with higher scores for selected BALB/c mouse MHC alleles (H-2Kd, H-2IAd). The Kolaskar and Tongaonkar scale was used to predict B cell epitopes in the IEDB server.

*Construction and evaluation of the multi-epitope polypeptides (polytopes)*

Selected epitopes were linked to each other using GGGS, KFERQ, GPGPG, HHAA, and HHAL linkers (35), to build the desired poly-epitope construction. S-tag oligopeptide sequence was also added to the C-terminal of the construct by the GGGS linker. Physicochemical parameters of the construct were investigated by the ProtParam server (https://web.expasy.org/protparam/). These parameters include molecular weight, instability index, aliphatic index, theoretical pI, amino acid composition, and *in vitro* and *in vivo* half-life. Also, polytope solubility was predicted using the SolPro server (http://scratch.proteomics.ics.uci.edu/explanation.html#SOLpro). Phyre2 (Protein Homology/analogY Recognition Engine V2.0) server (36) was used to assess the 3D structure of the IVP polytope. 

*Codon optimization of the IVP polytope in pET26b plasmid*

The Codon Adaptation Tool (JCAT) (37) was used for reverse translation and codon optimization of the IVP construct. The *NdeI* and *XhoI* restriction enzyme sites were added to 5’ and 3’ ends of the sequence, respectively. The DNA construct was then ordered to be cloned into the vector pET26b.

***IVP polytope immunogenicity experimental studies***

*Transformation of Escherichia coli BL21 (DE3) strain by vectors and expression of the polytope protein*

*E. coli *BL21 (DE3) cell was used as an expression host. It was transformed with IVP recombinant plasmid (pET26b) containing the poly-epitope construct and grown overnight at 37 °C on Luria Bertani agar (LB agar) medium. The transformed colonies were used for expression induction of the recombinant protein. To induce the expression of the recombinant polytope, 0.5M IPTG (Merck) was added and the culture was incubated for 4 hr. After that, the bacterial pellet was collected by centrifugation (4000 rpm, 10 min). 

*Confirmation of purified proteins by SDS-PAGE and western blotting*

Protein extraction was performed by protein lysis buffer (Tris 50 mM, Glycerol 50%, Triton x-100 0.1%, and PMSF 1mM, and 2λ of PMSF) and sonication. Expression was determined by SDS-PAGE (sodium dodecyl sulfate-polyacrylamide gel electrophoresis), so 20 μl of the sample with 10 μl of loading buffer (Bromophenol blue 0.25%, Glycerol 40%) was loaded on 12% SDS-PAGE gel. Coomassie blue stain (Merck) (0.25% (w/v) Coomassie blue R-250, 50% (v/v) methanol and 10% (v/v) acetic acid) was used for gel staining. Transmission of protein bands from gel to nitrocellulose membrane was carried out by transfer buffer in a western blot apparatus (APELEX) for one hour (38). Then, using the help of alkaline phosphatase-conjugated anti-S-tag antibody (Abcam, UK), and in the presence of the enzyme-substrate (NBT-BCIP) (Sigma), the desired protein band appeared.

*Protein purification by affinity chromatography*

Using the S-tag affinity chromatography the recombinant protein was purified. The prepared bacterial lysate was incubated together with the S-protein agarose for 1 hr in the column. After washing the unbound compounds, protein elution was performed with 3M magnesium chloride. Purified protein was dialyzed in PBS and its concentration was determined by the Bradford method. Purified protein confirmation was carried out by western blotting again.

*Preparation of chitosan and chitin microparticles for usage as adjuvants*

Choosing appropriate sizes of microparticles helps to stimulate a suitable outline of immune responses. Therefore, we evaluated poly epitope’s immunogenicity accompanied by chitin and chitosan microparticles (<40 μm) as adjuvants in both *in vivo* and *in vitro* experimental studies. To prepare small chitin and chitosan microparticles, pure chitin and chitosan powders (C-7170, C-3646, Sigma Chemical Co. St. Louis, MO) were suspended in sterile distilled water. The suspension was sonicated and sequentially filtered using 100-μm, 70-μm, and 40-μm filters (Cell Strainer, BD Falcon, Mexico). The chitin or chitosan microparticles (<40 μm) were obtained by centrifugation at 2800g for 10 min, the pellet was freeze-dried. The obtained powder was weighed and a suspension of 100 μg/100 μl concentration was prepared in distilled water, autoclaved, and stored at 4 °C until use. Particle sizes and size distributions were analyzed using a laser particle size analyzer (Malvern Master Sizer, Malvern Instruments, Ltd., Worcestershire, UK). More than 83% of the particles were <40 μm in diameter. The micro-particle suspensions were analyzed for the presence of endotoxin before use as adjuvants, using the Limulus Amebocyte Lysate (LAL) kit (Cambrex). The results showed that there was no endotoxin in the suspensions.

*Complex forming and immunization of BALB/c mice*

In compliance with all ethical principles of research on laboratory animals following the animal ethics guidelines of the Committee and the Code of Ethics [IR.SBMU.RETECH.REC.1396.1165], we chose thirty-five BALB/c female mice (6–8-week old) which were divided into 7 groups. Mice were managed in groups as follows: P+CsMP (50 µg protein+100 µg chitosan), P+CMP (50 µg protein+ 100 µg chitin), P (only 50 µg protein), CMP (100 µg chitin microparticles), CsMP(100 µg chitosan microparticles) and immunized in 40 µl volume intranasal (20 µl per nostril). The control group received 20 µl 1X sterile PBS alone and the sham group did not receive any composition. At an interval of 2 weeks, three immunizations were administered. Ten days after the third immunization, mice were bled and sacrificed by cervical dislocation.

***The assay of the inhaled animals’ immunity systems***

*Specimens collection*

The sera of experimental groups were collected from the blood and pooled to detect the antibodies and cytokines and also for neutralizing antibody response tests. The nasal washes were collected from mice. After sacrificing and cutting off mice’s lower jaws, a syringe needle was placed into the posterior opening of the nasopharynx and 0.5 ml PBS was used to wash the nasal cavity. Then collected nasal washes were centrifuged (700 g, 10 min.) and the supernatants were stored at -20 °C until used for IgA antibody assay. Next, the spleen was dissected out from each animal; B and T lymphocytes were isolated by Ficol 70 (Sigma) and then cultured in RPMI (Biosera) (with penicillin 100 u/mL and streptomycin 100 µg/ml) at a known density of cells for each later immunological assays. Proliferation stimulation of them (by antigen + adjuvant) was performed 24 hr after cell culture. Liver tissues were removed for examination of any pathological changes following the use of each complex formulation. H&E staining was consistent in all formalin-fixed slides. Liver tissues were kept in formalin until slide staining. Lung tissues and respiratory tracts were examined for color and size.

*Spleen lymphocytes proliferation assay (MTT assay)*

MTT (3-(4, 5-dimethylthiazol-2-yl)-2, 5-diphenyl tetrazolium bromide) assay was performed according to the standard protocol (39). In brief, 100 µl phenol red and serum-free RPMI medium containing 40000 lymphocytes were plated in each well of a 96-well plate. After 24 hr incubation at 37 °C, 10 µg of IVP polytope and 10 µg of each polymer (CMP or CsMP) were added to each well to stimulate the proliferation of lymphocytes. As a positive control, cells were stimulated with 2.5 μg/ml of phytohaemagglutinin (PHA), and PBS was added to wells as the negative control. After 48 hr, 100 μl of MTT solution (5 mg/ml) was added per well. After 4 hr of incubation, colored crystals of formazan were dissolved with 100 μl of isopropanol. Optical density (O.D.) was read on an ELISA reader at 570 nm after 5 min. The mean values of the proliferation index (PI) with antigens were calculated for samples. The geometric mean values of the antigen-stimulated triplicates were calculated and distributed with the geometric mean values of the medium control triplicates to obtain the proliferation index (PI).

*ELISA (Enzyme-linked immune sorbent assay)*

Serum IgG2a (eBioscience™ Mouse IgG2a ELISA Ready-SET-Go! ™ Kit), serum and nasal wash total IgA (Bioassay Technology Laboratory Mouse immunoglobulin A ELISA kit), and cytokine concentrations secreted into the supernatants from stimulated splenocytes were quantified using ELISA kits for IL-6, TNF-α, IL-10, and IL-17 according to the manufacturer’s (R&D Systems, DuoSet® ELISA development system, USA) instructions. Briefly, ELISA plates were coated with the capture antibody. After washing, plates were blocked by blocking solution. Then, 1/2 dilution of sera, culture supernatants, and antibody standards were added to the wells, incubated for 2 hr. A detection antibody was used after washing and the conjugated enzyme was added. Twenty min after adding the substrate solution, a stop solution was used and the plate’s ODs were read by ELISA reader at 450 nm. The antibody concentrations were calculated using the standards and linear regression of the log-transformed readings. 

***IFN-γ ELISPOT assay***

According to the instructions of the Mouse IFNg ELISPOT kit (eBioscience), the ELISPOT procedure was carried out in triplicate for each of the experimental groups. In brief, a 96-well PVDF plate (Millipore) was coated with a capture antibody (anti-mouse IFN-γ) solution and incubated at 4 °C overnight. The plate was washed 2 times and blocked with complete RPMI-1640 for one hour at room temperature. Then 3×10^6^ spleen lymphocytes were added to each well and stimulated with 10 or 20 µg of the IVP polytope and adjuvants. Positive controls were stimulated by PHA. Plate was incubated at 37 °C in 5 % CO_2_ for 48 hr. Cells were then removed by wash buffer and the biotin-conjugated detection antibody was added to the wells and incubated for 2 hr at room temperature. The plate was washed 4 times with wash buffer and Avidin-horseradish peroxidase solution was added and incubated for 45 min. After washing five times the substrate solution was added to each well and the plate was kept in the dark, up to 60 min for spot development. Then the plate was washed 3 times with distilled water. It dried at room temperature. Spots were counted by a loop and the results were calculated as the number of spot-forming cells per 300 thousand cells.

***Virus neutralization assay***

This procedure was performed in an isolated *influenza* laboratory at Pasteur Institute of Iran. Animal sera were heat-inactivated (56 °C, 30 min) before diluting serially 2-fold by culture medium (dilutions: 1 to 1/256). Each diluted serum (50 µl) was mixed with 50 µl 100 TCID_50_ of each H1N1 and H3N2 virus. The virus-serum mixtures were incubated for 1 hr at room temperature. Then they were inoculated to the MDCK (Madin-Darby Canine Kidney) cell layer previously cultured in a 90 % confluent 96-well plate. After 48 hr (for H1N1 virus) and 72 hr (for H3N2 virus) incubation, the cytopathogenic effects (CPE) of each well were assessed by an inverted microscope. The supernatants were added to an equal volume of 0.5% chicken red blood cells and kept for 1 hr (at room temperature) in a 96-well plate. The absence of hemagglutination was considered neutralization. 

***Toxicity evaluation of IVP poly-epitope***

Liver tissues as the sensitive organ to the toxins were fixed with 10% neutral buffered formalin for histopathology. After fixation, sections were embedded in paraffin and stained with Hematoxylin & Eosin. The histopathological examination was performed as described in Cui *et al*. study (40). Also, during surgery, the size and color of the lungs and respiratory tracts of mice were studied. 

***Statistical analysis***

Data are expressed as the mean ± standard error of the mean (SEM), and statistical analysis was performed using ANOVA (analysis of variance) with Tukey’s *post hoc* test. (* *P*<0.05, ** *P*<0.01, *** *P*<0.001).

## Results

***Evaluation of polytope protein***

*Protein sequence retrieval and predicted epitopes*

Hemagglutinin (HA), nucleoprotein (NP), and M2 proteins were selected (Table 1) for immunogenic epitope mapping. The related sequences of these proteins were retrieved, then each protein was studied in the UniprotKB database in terms of its topology of structure, as well as its post-translational modifications. We considered haplotype “d” as a dominant allele in the BALB/c mice population and predicted MHC I and II restriction epitopes in the IEDB server. Tables 2, 3, and 4 show the selected epitopes from each prediction process. 

*Construction of the multi-epitope polypeptide and 3D structure prediction*

**Figure 1:**
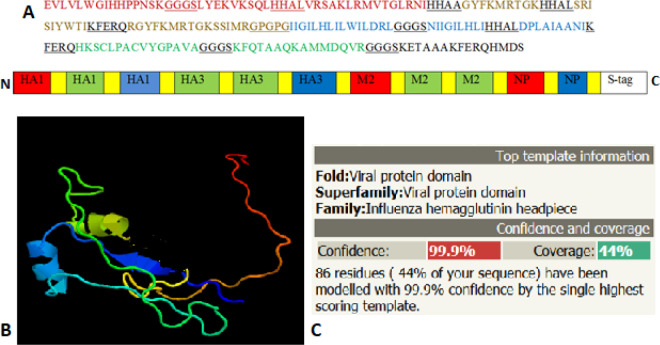
Schematic representation of the IVP poly-epitope. A) Arrangement of predicted epitopes from selected proteins is shown; the yellow segments represent the underlined linkers in sequence. B) The Phyre2 protein model with 99.9% confidence with influenza hemagglutinin headpiece (C)

Based on IEDB server predictions, epitopes from the aligned consensus sequences of the proteins with higher scores were chosen (Tables 3 and 4). *Influenza *virus epitopes fused with proper linkers as shown in Figure 1. 

The selected peptides were connected with flexible and immune stimulator linkers (Figure 1) and the theoretical 3D model of the IVP construct was carried out using Phyre2 protein fold recognition server (Job ID: C598bb52f9c6f624). 

The physicochemical properties of the polypeptide are demonstrated in Table 5. Based on Solpro server results, the IVP protein predicted soluble upon overexpression with a probability of 0.90. Based on our *in silico* structural and physicochemical evaluations, the polypeptide was predicted a suitable vaccine candidate. 

Reverse translation and codon optimization were carried out simultaneously by the JCAT server. CAI-Value (Codon Adaptation Index) of the improved sequence for the construct was 1.0 and the GC-content of the improved sequence for IVP polypeptide was 51.60. The *NdeI* and *XhoI *restriction sites were added to 5’ and 3’ ends of the sequence and designed in the pET26b expression vector. 

***Confirmation of protein by Western blotting***

**Figure 2:**
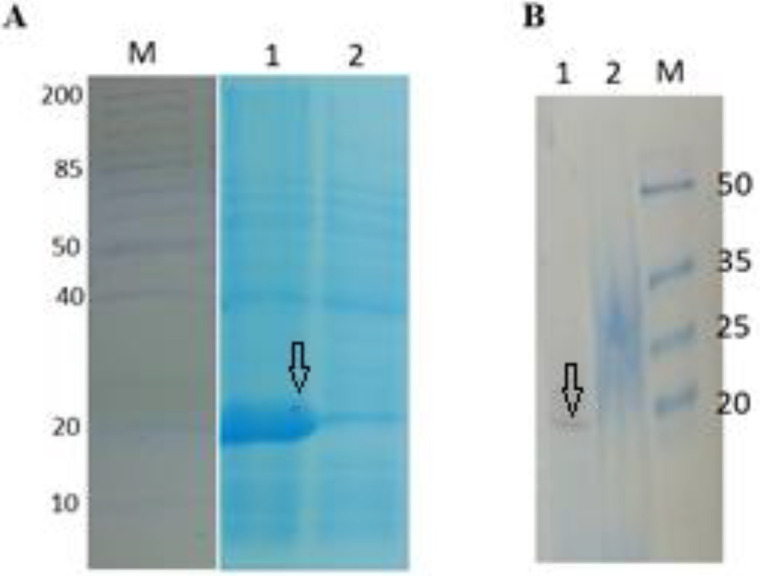
Influenza virus poly-tope protein (IVP) expression analysis. (A) SDS-PAGE (12%) followed by an R250 Blue Coomassie stain. (B) Confirmation of the protein expression by western blotting. M: Protein Molecular Weight Marker (KDa), Lane 1: poly-tope protein, Lane 2: Non-induced controls

After transformation of *E. coli *BL21 (DE3) by vectors and expression induction of the polytope, the protein was extracted and purified by S-tag affinity chromatography. The size and purity of it were confirmed by SDS-PAGE. Polytope (~ 20 KDa) expression has been shown in Figure 2.

***Evaluation of animals’ immune systems***

*The proliferation assay spleen lymphocytes by MTT*

**Figure 3:**
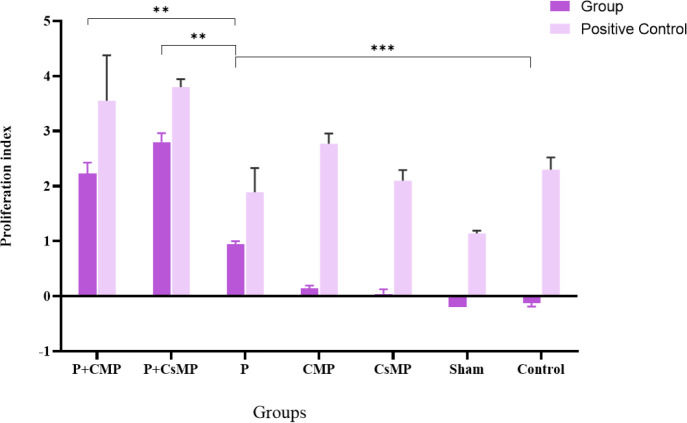
Elicited proliferative response after* in vitro *sensitization of the splenic cells by polytopes and adjuvants. Significantly higher cell proliferation index was observed for two P+CsMP and P+CMP groups. (* *P*<0.05, ** *P*<0.01, *** *P*<0.001)

Cellular response stimulation has been indicated by elevated splenic cell expansion. Proliferation index (PI) was calculated by [O.D. of test sample−O.D. of negative control /O.D. of negative control]. The mean values of the proliferation index in the P+CsMP group were obtained as 2.8±0.36 (Figure 3). Positive controls in stimulation with PHA in each group showed a normal condition of cells in culture. 

*Cytokines and antibodies analysis by ELISA and IFN-γ ELISPOT*

**Figure 4:**
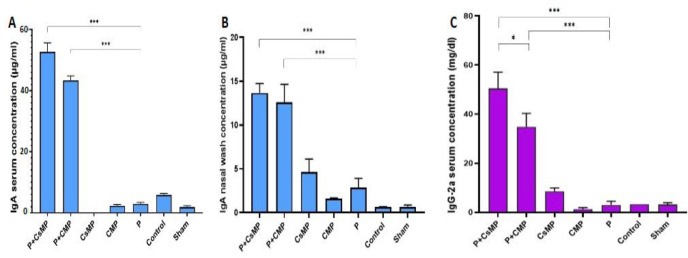
Production of the antibodies in mice following intranasal administration of polytope mixed with adjuvants. The ELISA assay was performed on pooled sera from five mice in each group. A) Serum IgG-2a, B) Serum IgA, and C) Nasal wash IgA concentrations. (* *P*<0.05, ** *P*<0.01, *** *P*<0.001). As shown, the immunized mice in P+CsMP and P+CMP groups produced significant (*P*<0.001) IgG2a antibody response compared with the P group. The P+CsMP group had a higher concentration of this antibody than the P+CMP group (*P*<0.05). Similar observations were also observed in the nasal wash and serum production of IgA in animals

IgA and IgG2a antibody concentrations in mice sera were shown in Figures 4A and C. The assay has been performed on pooled sera from five mice in each group. IgA concentration in nasal washes was also determined by ELISA assay (Figure 4B). 

**Figure 5:**
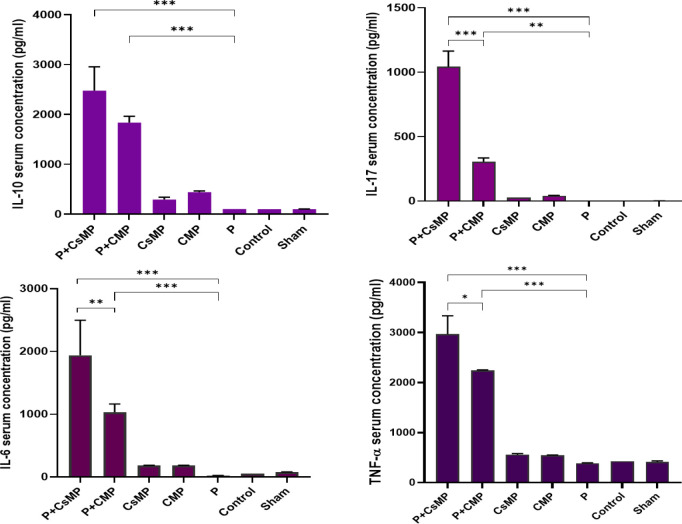
Serum cytokine concentration analysis in mice following the intranasal administration of polytope mixed with adjuvants after 3-times immunization. Pooled sera from five mice in each group, IL-10, IL-17, IL-6, TNF-α serum concentrations measured by ELISA. (* *P*<0.05, ** *P*<0.01, *** *P*<0.001). P+CsMP and P+CMP groups showed high IL-6, TNF- α, IL-10, and IL-17 cytokine concentrations. There were observed upper cytokine concentrations in the P+CsMP groups

**Figure 6:**
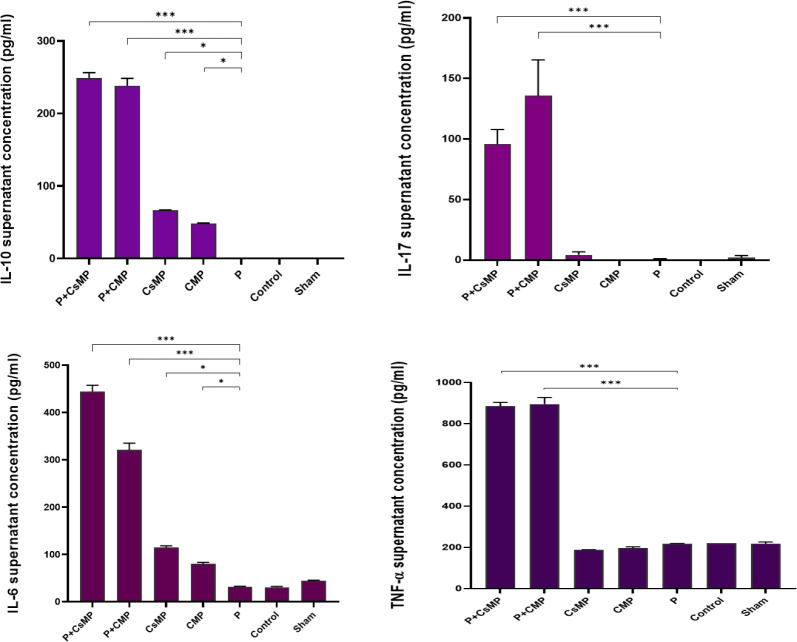
Cytokine concentrations in the supernatants of spleen cells. Isolated splenocytes (2×10⁵) were cultured and stimulated with 10 µg of the construct plus adjuvants. 48 hr after* in vitro* sensitization by polytopes and adjuvants, secreted cytokine concentrations (IL-6, TNF-α, IL-17, and IL-10) in the supernatant of splenocytes were presented. (* *P*<0.05, ** *P*<0.01, *** *P*<0.001)

Similarly, the concentration of secreted cytokines (IL-6, IL-10, IL-17, and TNF-α) in the serum and the supernatants of splenocytes after exposure to polytopes was shown in Figures 5 and 6. 

**Figure 7:**
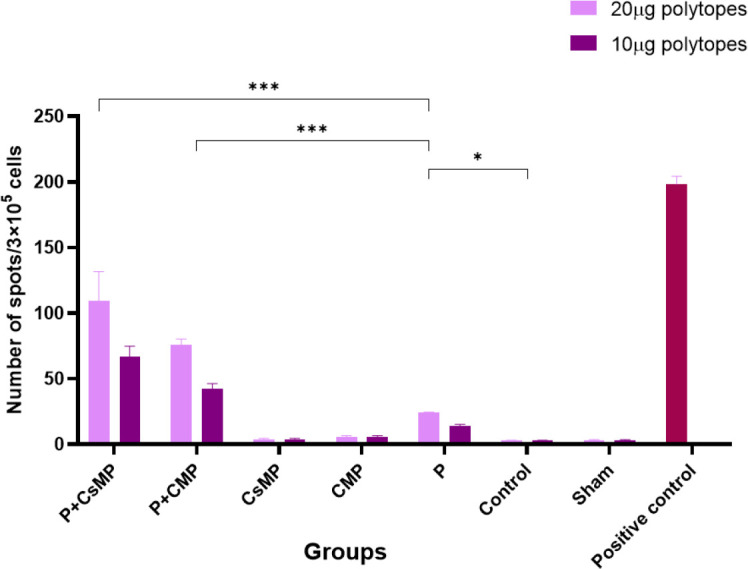
IFN-γ ELISPOT assay. Cell-mediated immunity was assessed by detection of spots of IFN-γ secreting splenic cells stimulated by 10 µg or 20 µg of polytopes in culture. The mean number of spots was calculated in the triplicate wells. (* *P*<0.05, ** *P*<0.01, *** *P*<0.001). The spot number of the P group for both polytope concentrations was more than CsMP alone, CMP alone, sham, and control groups (*P*<0.05) but not more than P+CsMP and P+CMP groups (*P*<0.001)

Cell-mediated immunity was assessed by detection of spots of IFN-γ secreting splenic cells stimulated by 10 µg or 20 µg of polytopes in cultures. The mean number of spots was calculated in the triplicate wells. In both polytopes concentrations, we observed a significant increase in the number of spots for P+CsMP and P+CMP groups compared with other groups (Figure 7).

***Virus neutralization test***

**Figure 8.**
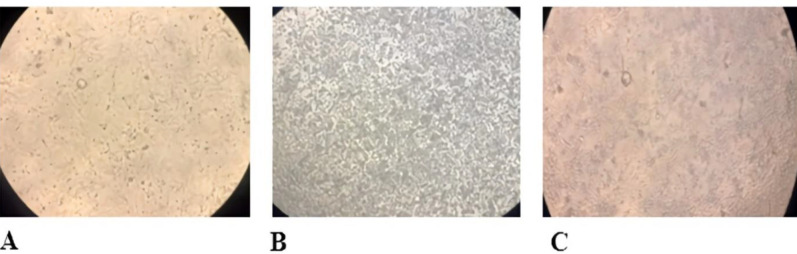
Cytopathic effect (CPE) on virus inoculated MDCK cells. A) Uninfected and normal MDCK cells (as negative control), B) Infected cells with virus proliferation caused by CPE (as positive control), C) P+CsMP treated group showed normal MDCK cells

To assess the antibodies detected by ELISA assay that could protect cells against viral infection *in vitro*, the MDCK cells were inoculated with a mixture of serum dilutions and *influenza* viruses. As shown in Figure 8, in 1:16 serum dilution for both P+CsMP and P+CMP groups, antibodies were able to neutralize the H1N1 virus and inhibit its propagation in the MDCK cell line. For the H3N2 virus, in serum dilution 1:256 for the P+CsMP group and 1:128 for the P+CMP group, antibodies had this capacity. Also, the cytopathic effect on MDCK cells monolayer was evaluated as shown in Figure 8. 

***Microscopic evaluation of the polytope effects***

**Figure 9:**
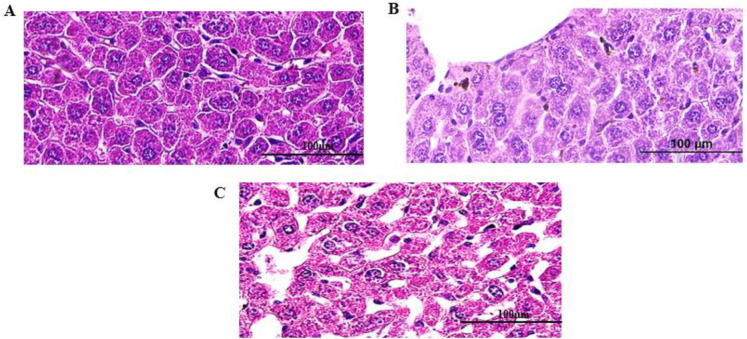
Microscopic view of the Hematoxylin & Eosin stained liver tissue sections (Bar= 100μm). There are no specific pathological changes in liver tissues of immunized mice with A) P+CsMP, B) P+CMP, and C) control groups

Histological examination of the liver tissues showed (Figure 9) completely healthy hepatocytes and tissue structure. There were observed no signs of any tissue damage and significant pathologic changes in any treated groups as well as the control group. The lung tissue and respiratory tract were normal in the macroscopic study.

## Discussion

To create an effective cocktail vaccine formulation (based on immune-dominant epitopes (41) and appropriate adjuvants eliciting both CTL and Th immune responses), we chose virulence proteins based on their functions and roles during infection from *influenza *H1N1 and H3N2 viruses to epitope mapping. After construction of the multi-epitope polypeptide from predicted epitopes, based on our *in silico* structural and physicochemical evaluations, the polypeptide was predicted a suitable vaccine candidate. In addition to ease of use and suitable physicochemical and anatomical conditions of the nasal cavity for the development of inhalation vaccines, stimulating the appropriate mucosal responses such as secretion of IgA antibody and cell-mediated immunity justifies the need for this type of vaccine investigation. Simulation of a natural infection is made possible by creation of inhaled vaccines. As the thin and permeable nasal mucosal surface is the route for respiratory pathogen entrance, their vaccines also can be delivered through this direction. So, they can elicit immune responses at the pathogen’s entry site (as well as systemic responses) and help to eliminate infection transmission.

From the proliferation stimulation assay of splenocytes, it can be concluded that the combination of chitin and chitosan with polytopes effectively enhanced proliferation as compared with polytopes alone; so these polymers have offered a further boost in proliferation index for the two P+CsMP and P+CMP groups. In our study, cell proliferation was accompanied by increased concentration of IL-6 and TNF-α cytokines in sera and supernatant of splenocytes. Considerably cytokine production was significantly higher for the groups being treated with P+CsMP and P+CMP but not for those being treated with CsMP, CMP, or P only. Alongside these results, the IFN- γ ELISpot assay revealed that P+CsMP and P+CMP groups indicatively had increased numbers of IFN-γ-secreting cells compared with the rest of the groups. IFN-γ was secreted in response to the re-activation of cells *in vitro*. IFN-γ is generally produced by dendritic cells, Th1, and cytotoxic T lymphocytes. IL-6 elevation causes T cell differentiation and growth, and inhibition of lymphocyte apoptosis (17).

Upper levels of IFN-γ producing cells and TNF-α secretion in sera and supernatants observed in our main study groups confirmed other reports that showed chitosan and its derivatives could have overexpressed such cytokines in the mouse models (42-43). Increased IFN-γ induces antibody class switching to IgG2a.

Our findings showed that intranasal administration of IVP adjuvanted protein resulted in increased antibody (humoral) immune responses in mice, as demonstrated by enhanced IgG2a and IgA concentrations in their sera and IgA concentration in the nasal wash. It is well known that the IgG2a antibody is indicative of the Th1 response that accelerates pathogen clearance (25). IgA antibodies can entrap pathogens at their local invasion. In addition to inhibiting pathogen growth in the lower respiratory tract, there is a need for antibodies and responses in the upper respiratory tract to protect against pathogen entry. Therefore, inhalation vaccines against respiratory pathogens should not only reduce the severity of the disease by induction of systemic humoral responses but also should inhibit the pathogen at the site of entry with the help of secretory IgA antibodies.

Furthermore, we get an increase in the concentration of IL-10 in P+CsMP and P+CMP groups. It was shown that chitin (44) and chitosan (45) binding to the surface receptors of macrophages leads to production of interleukin-10. It is demonstrated that there is a relationship between the size of chitin microparticles and the production of interleukin-10 cytokine. Macrophages produced both IL-10 and TNF-α through the dectin-1 receptor and the Syk signaling pathway when they were stimulated by microparticles below 40 μm (often 2-10 μm) in size, but produced TNF-α alone by the TLR-2 pathway in response to microparticles with a size of 40-70 micrometers (46).

Increased concentrations of IL-17 have been previously reported in the intranasal administration of vaccines (47). Chitin particles induce IL-17 amplification by activation of the TLR-2-MyD88 signaling pathway (48). IL-17 response contributes to mucosal immunity increasing secretory IgA transportation to the nasal cavity (49)in addition to causing neutrophilic inflammation in mice, mediated a pronounced influx of CD19(+. The augmented local and systematic IgA concentrations in P+CsMP and P+CMP groups are justified by the presence and function of Th17 cells, which is also confirmed by increased IL-17 concentration in sera and supernatants of mentioned groups. It is worthy to formulate vaccines prompting IL-17 responses. The results suggested that small-sized chitin microparticles had a strong potential to increase both cellular and humoral immune responses and elicited a balanced Th1/Th2/Th17 response and that these microparticles may be a safe and efficacious adjuvant candidate suitable for a wide spectrum of vaccines.

By mucoadhesive property, chitin and chitosan cause antigen entrapment in mucosal tissue and so enhance their uptake by nasal-associated lymphoid tissue (40). Their capability to depot and slowly release antigens increases the presentation of APCs and produces more potent responses. 

Our evaluation showed immunized mice in P+CsMP and the P+CMP groups produced significant (*P*<0.001) IgG2a antibody response compared with the P group. The P+CsMP group has a higher concentration of this antibody than the P+CMP group (*P*<0.05). Similar observations were also observed in the nasal wash and serum production of IgA in animals. CsMP, CMP, and P alone groups elicited low levels of each of the cytokines. In contrast, P+CsMP and P+CMP groups showed high IL-6, TNF- α, IL-10, and IL-17 cytokine concentrations. Upper cytokine concentrations were observed in P+CsMP groups for both sera and supernatant samples except for the supernatant concentrations of IL-17 and TNF- α which were slightly higher in the P+CMP group. 

Some studies showed that the immune-potentiating effects of chitin and chitosan in living models vary in terms of quantity and quality (42-46). In contrast with those studies, our results showed magnitude elevation of antibodies and cytokines as well as lymphocyte proliferation (not significant for all of them) to chitosan micro-particle than to chitin micro-particle. It can be concluded that combination of chitin and chitosan with polytopes effectively enhanced proliferation as compared with polytopes alone so these polymers have offered a further boost in proliferation index for the two P+CsMP and P+CMP groups.

This difference may be due to the route of vaccine administration (intranasal) in our study and/or the fact that chitosan has more positive charges that lead to higher affinity for the negatively-charged poly-peptides and mucus, so depot and presentation of antigen to the immune system improves. It is shown that the cellular immune responses can be elicited via cross-presentation of antigens through MHC I molecules in APCs by chitosan. For the reason that the protonated amino groups of chitosan presented in lysosomes after absorption to the APCs, antigens can be presented as endogenous antigens when lysosome disruption occurs following the influx of water and ions from the lysosome (25). For designing safe and efficient mucosal vaccines, chitin and chitosan microparticles are valuable candidates as they can properly overcome chemical and physical mucosa barriers (50).

The results of VNT (virus neutralization test) showed the protection of MDCK cells against viral infection *in vitro*. The VNT is a precious assay for assessment of the humoral response following *influenza* vaccination or natural infection (51). The presence of seroprotective antibodies against *influenza* viruses in the sera was confirmed by this test. We found that mice in P+CsMP and P+CMP groups had satisfactory neutralizing antibodies compared with the control group against both H1N1 and H3N2 virus subtypes. Antibodies against the H3N2 virus in the P+CsMP group could inhibit virus entry and propagation *in vitro* 16 times higher than antibodies against the H1N1 virus in this group. For the P+CMP group, antibodies against the H3N2 virus could inhibit this virus 8 times higher than antibodies against the H1N1 virus in this group. Overall, our results showed higher induction of neutralizing antibodies against the H3N2 virus compared with the H1N1 virus.

In summation, since the mucosal surface area of the nasal cavity is large and the nasal-associated lymphoid tissue is the tissue that generates local responses (52), mucosal immunity can help to break the disease transmission chain, and designing inhalation vaccines with suitable adjuvants such as chitin and chitosan biopolymers is highly desirable. Chitosan and chitin micro-particles can carry and release antigens accurately to the desired sites, for instance to the intestinal Peyer’s patches in oral usage (53) or the nasal-associated lymphoid tissue in the nasal usage. By amine groups in chitin and chitosan structures, they can adhere to the mucus (54), then attach to the β-integrins at the epithelial cell surfaces, causing them to cluster and cladin-4 proteins to enter into the cells, leading to the opening of the tight junctions (55). Chitosan and chitin with the mucoadhesive property and by tri-functional feature (adjuvant/carrier/ immune-stimulant) can provide satisfactory mucosal immunity. Chitosan and chitin are capable of long-term depot and storage of antigens at the site of administration. By having positive charges (especially chitosan), they can bind to peptides (as vaccine antigens) by electrostatic interactions, reduce the rapid removal of antigens from the nasal cavity, keep antigens in the mucosa for a longer period, and present them more efficiently to antigen-presenting cells (24).

The results support the fact that immunogenic poly-tope protein in the presence of chitin and chitosan microparticles as mucosal adjuvants is able to induce mucosal and systemic antibody and cell-mediated responses in BALB/c mice. This study provides an outline for investigation of the same formulations for human-effective inhaled vaccines.

**Table 1:** Chosen virulence proteins from influenza virus type A for epitope prediction procedures

**Protein name**	**PDB code**
**Hemagglutinin H1N1(HA1)**	P03452
**Hemagglutinin H3N2(HA3)**	P03437
**Matrix protein 2(M2)**	P06821
**Nucleoprotein (NP)**	P03466

**Table 2:** Chosen high score B cell linear epitopes from *influenza virus *proteins

**Antigenic protein**	**Position**	**Peptide Sequence**	**Score**
**HA1**	188-202	EVLVLWGIHHPPNSK	1.125
**NP**	272-286	HKSCLPACVYGPAVA	1.179
**M2**	32-46	IIGILHLILWILDRL	1.123

**Table 3:** Selected MHC-I binding peptides from *influenza virus *proteins

**Protein**	**Position**	**Peptide sequence**	**MHC allele**	**Percentile rank**
**HA1**	461-469	LYEKVKSQL	H-2-Kd	0.2
**HA3**	272-280 244-252	GYFKMRTGK SRISIYWTI	H-2-Kd	0.4 0.5
**M2**	24-32 31-39	DPLAIAANI NIIGILHLI	H-2-Kd	2.8 3.2

**Table 4:** Selected MHC-II binding peptides from *influenza virus *proteins

**Protein**	**Position**	**Peptide sequence**	**MHC allele**	**Percentile rank**
**HA1**	323-337	VRSAKLRMVTGLRNI	H-2-IAd	1.84
**HA3**	271-285	RGYFKMRTGKSSIMR	H-2-IAd	3.21
**NP**	229-243	KFQTAAQKAMMDQVR	H-2-IAd	0.59

**Table 5.** Physicochemical parameters of IVP protein

**Physicochemical parameters**	**IVP protein**
**Number of amino acids**	197
**Molecular weight(Da)**	21682.39
**Theoretical pI**	10.45
Extinction coefficients/Abs0.1% (=1 g/l)	24075/1.110
**Instability index**	38.89 stable

IVP: Influenza virus poly-tope protein

## Conclusion

Our designed protein named IVP accompanied by chitin or chitosan showed enhanced serum and nasal antibodies in addition to cellular and humoral responses. An increase in the number of IFN-γ-producing cells in re-stimulation of splenocytes in the culture medium by poly-tope as well as rise in the concentrations of IL-6, IL-17, and TNF-α along with the regulatory response of IL-10, presented the capacity of constructs to provoke immune responses. The neutralization test ultimately confirmed the high efficacy of the protein in inhibiting the virus. 
